# Pilot Study of *CYP2B6* Genetic Variation to Explore the Contribution of Nitrosamine Activation to Lung Carcinogenesis

**DOI:** 10.3390/ijms14048381

**Published:** 2013-04-16

**Authors:** Catherine A. Wassenaar, Qiong Dong, Christopher I. Amos, Margaret R. Spitz, Rachel F. Tyndale

**Affiliations:** 1 Department of Pharmacology and Toxicology, The University of Toronto, Toronto, ON M5S 1A8, Canada; E-Mail: catherine.wassenaar@utoronto.ca; 2 Department of Epidemiology, The University of Texas M.D. Anderson Cancer Center, Houston, TX 77030, USA; E-Mail: qdong@mdanderson.org; 3 Department of Community and Family Medicine, Geisel School of Medicine, Dartmouth College, Hanover, NH 03755, USA; E-Mail: christopher.i.amos@dartmouth.edu; 4 Department of Molecular and Cellular Biology, Baylor College of Medicine, Houston, TX 77030, USA; E-Mail: spitz@bcm.edu; 5 Campbell Family Mental Health Research Institute, Centre for Addiction and Mental Health, Departments of Psychiatry, Pharmacology and Toxicology, the University of Toronto, Toronto, ON M5S 1A8, Canada

**Keywords:** CYP2B6, CYP2A6, CHRNA5-CHRNA3-CHRNB4, tobacco specific nitrosamines, lung cancer risk, genetic variation

## Abstract

We explored the contribution of nitrosamine metabolism to lung cancer in a pilot investigation of genetic variation in *CYP2B6*, a high-affinity enzymatic activator of tobacco-specific nitrosamines with a negligible role in nicotine metabolism. Previously we found that variation in *CYP2A6* and *CHRNA5-CHRNA3-CHRNB4* combined to increase lung cancer risk in a case-control study in European American ever-smokers (*n* = 860). However, these genes are involved in the pharmacology of both nicotine, through which they alter smoking behaviours, and carcinogenic nitrosamines. Herein, we separated participants by *CYP2B6* genotype into a high- *vs.* low-risk group (*1/*1 + *1/*6 vs. *6/*6). Odds ratios estimated through logistic regression modeling were 1.25 (95% CI 0.68–2.30), 1.27 (95% CI 0.89–1.79) and 1.56 (95% CI 1.04–2.31) for *CYP2B6*, *CYP2A6* and *CHRNA5-CHRNA3-CHRNB4*, respectively, with negligible differences when all genes were evaluated concurrently. Modeling the combined impact of high-risk genotypes yielded odds ratios that rose from 2.05 (95% CI 0.39–10.9) to 2.43 (95% CI 0.47–12.7) to 3.94 (95% CI 0.72–21.5) for those with 1, 2 and 3 *vs.* 0 high-risk genotypes, respectively. Findings from this pilot point to genetic variation in *CYP2B6* as a lung cancer risk factor supporting a role for nitrosamine metabolic activation in the molecular mechanism of lung carcinogenesis.

## 1. Introduction

Variation in genes participating in nicotine and nitrosamine pharmacology has been associated with lung cancer risk among smokers. We have reported that variation in the nicotine and nitrosamine metabolizing gene, *CYP2A6*, and the nicotinic receptor subunit gene cluster, *CHRNA5-CHRNA3-CHRNB4 (CHRNA5-A3-B4)*, combined to increase cigarette consumption and lung cancer risk among European American cigarette smokers [1]. Owing to the contribution of these genes to nicotine pharmacology, *CYP2A6* and *CHRNA5-A3-B4* increase lung cancer risk indirectly through increased cigarette consumption and thus increased carcinogen exposure. Additionally, these genes could be contributing to lung cancer risk directly through altered nitrosamine pharmacology—the CYP2A6 enzyme metabolically activates tobacco specific nitrosamines (TSNA) [2] and the nicotinic receptors are high-affinity binding sites for nitrosamines [3].

To explicitly explore the potential contribution of altered nitrosamine pharmacology to lung cancer risk among smokers, we have extended our investigation of gene variants to *CYP2B6*. CYP2B6, like CYP2A6, is expressed in the lung in addition to the liver [4,5] and metabolically activates TSNAs to DNA-reactive products [2,6]. Enzymatic activation of the potent lung procarcinogenic TSNA, 4-(methylnitrosamino)-1-(3-pyridyl)-1-butanone (NNK), has been linked to NNK-induced lung tumors [7], and the CYP2B6 enzyme has a high affinity for NNK [8]—roughly ten-fold higher compared to CYP2A6 [6]. CYP2A6, on the other hand, has a higher affinity for the TSNA, *N*′-nitrosonornicotine (NNN), than for NNK [6,9] and may preferentially metabolize NNN while CYP2B6 preferentially metabolizes NNK. In contrast to CYP2A6, CYP2B6 has a low affinity for nicotine [8] and makes a minor contribution to systemic nicotine metabolism (<20%) and characterized gene variants are not associated with differences in cigarette consumption [10,11]. Consequently, *CYP2B6* gene variants are biologically positioned to influence the risk of developing lung cancer among cigarette smokers through altered metabolic activation of nitrosamines (potentially within the lung) as opposed to indirectly through cigarette consumption.

Here, we present a feasibility study of genetic variation in *CYP2B6* as a potential lung cancer risk factor. To propose a role for *CYP2B6* gene variants and altered nitrosamine activation, it was critical to assess whether *CYP2B6* operated independently of *CYP2A6* to influence lung cancer risk among smokers. The *CYP2B6* and *CYP2A6* genes are located in proximity on chromosome 19q23 with their 5′ regulatory regions oriented towards each other [12,13], and reports of genetic linkage, co-regulation and correlated liver expression levels [11–14] coupled with the common role of these enzymes as activators of TSNAs suggested a potential redundant role of *CYP2B6* and *CYP2A6* gene variants. In this exploratory analysis, our main objectives were to estimate the effect size of *CYP2B6* relative to *CYP2A6* and *CHRNA5-A3-B4* and to establish a probable independent contribution of *CYP2B6* to the risk of lung cancer among smokers and in so doing implicate a role for altered nitrosamine activation in the molecular mechanism of lung carcinogenesis.

## 2. Results and Discussion

*CYP2B6* genotype frequencies did not deviate from the Hardy-Weinberg equilibrium overall, in controls or in cases. We found the CYP2B6*6 haplotype and the CYP2B6*4 and *9 alleles at a frequency of 24%, 2.5% and 0%, respectively, in control subjects, which is consistent with other study samples of European descent [15,16]. Less than five percent (*n* = 41) of the 860 participants from the original study possessed the CYP2B6*4 allele in isolation precluding an independent investigation of this allele, and thus were excluded from analyses. Table 1 summarizes the characteristics and CYP2B6*6 genotyping results of the 819 study participants included in analyses.

To assess the genetic risk for developing lung cancer, participants were separated into a high- and low-risk genotype group for each gene. We employed the same high- *vs.* low-risk lung cancer genotype groups for *CYP2A6* and for *CHRNA5-A3-B4* as before (Table 1): *CYP2A6* normal *vs.* reduced metabolizers (those who possess at least one reduced activity allele) and *CHRNA5-A3-B4* AA *vs.* GG/GA [1]. For *CYP2B6*, the CYP2B6*6 haplotype appeared to act recessively whereby only those homozygous for the CYP2B6*6 allele were afforded protection from lung cancer risk. Hence, participants with the CYP2B6*1/*1 and CYP2B6*1/*6 genotypes were pooled together into the high-risk group, while participants with the CYP2B6*6/*6 genotype comprised the low-risk group. The effect of the CYP2B6*6 variant allele relative to the CYP2B6*1 wildtype allele on the enzymatic activation of TSNAs has not yet been characterized. Our findings appear to be similar to the effects of CYP2B6*6 on the well-characterized CYP2B6 substrate efavirenz where a reduction in *in vitro* and *in vivo* metabolism and a higher incidence of side effects were only evident with the CYP2B6*6/*6 genotype [17–19]. The CYP2B6*6 allele is associated with reduced protein expression [11,20]—perhaps a substantial reduction in CYP2B6 protein (*i.e.*, CYP2B6*6/*6) is necessary to influence lung cancer risk.

**Figure 1:**
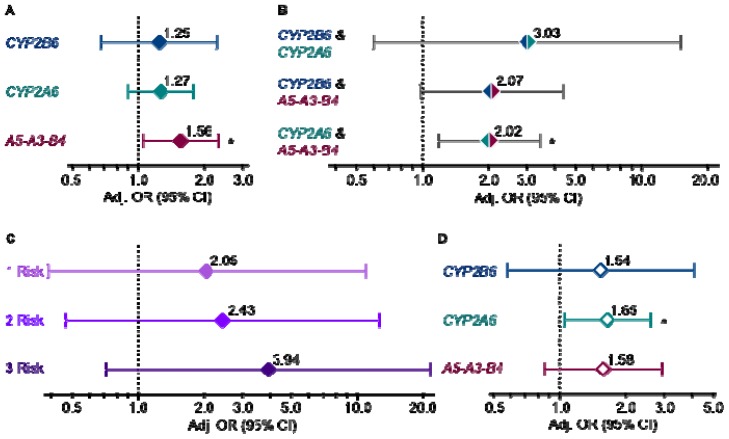
(**a**) Lung cancer risk by *CYP2B6*, *CYP2A6* and *CHRNA5-A3-B4* genotype group for each gene alone (*p* = 0.47, 0.17 and 0.03); (**b**) each gene pair in combination (*p* = 0.18, 0.06 and 0.01); (**c**) all three genes in combination where participants were classified as having 0, 1, 2 or 3 high-risk genotypes and those with 0 high-risk genotypes served as the reference group (*p* = 0.40, 0.29 and 0.11); and (**d**) in the lighter-smoking stratum, cigarettes per day ≤ 20 (*p* = 0.39, 0.03 and 0.14). All odds ratios were determined by logistic regression modeling and adjusted for age, gender and log pack-years. Genotype comparisons were as follows: CYP2B6 *1/*1, *1/*6 vs. *6/*6, *CYP2A6* normal *vs*. reduced metabolizer, *CHRNA5-A3-B4* AA *vs*. GG/GA. *p ≤ 0.05. Refer to Supplementary Tables S1–S4 for subject numbers and unadjusted and adjusted lung cancer odds ratios for Figure 1A–D, respectively.

Figure 1A presents lung cancer odds ratios for each gene alone with the low-risk genotype group serving as the reference group. Of note, the odds ratios for *CYP2B6* and *CYP2A6* were of similar magnitude, although the associations did not reach statistical significance. The odds ratio conferred by the CYP2B6*6 genotype is unlikely to be driven through an indirect association with *CYP2A6* gene variants: we found no significant correlations between *CYP2A6* and *CYP2B6* gene variants (*r^2^* = 0.00–0.01 for each pairwise comparison) when we investigated linkage disequilibrium using Haploview (v4.2) [21]. Furthermore, when we concurrently evaluated all three genes in a multivariate logistic regression model there was a negligible shift in the odds ratio for each gene (1.25 to 1.25 for *CYP2B6*; 1.27 to 1.26 for *CYP2A6*; 1.56 to 1.55 for *CHRNA5-A3-B4*) suggesting that variation in each gene is contributing independently to the risk of lung cancer.

Combined gene analyses provided additional evidence that *CYP2B6* was contributing independently to cancer risk. We first evaluated each gene pair by comparing participants possessing both high-risk genotypes to those with both low-risk genotypes (Figure 1B). For example, individuals in both the *CYP2B6* and *CYP2A6* high-risk genotype groups (CYP2B6 *1/*1, *1/*6 and *CYP2A6* normal metabolizer) were compared to those in both low-risk genotype groups (CYP2B6 *6/*6 and *CYP2A6* reduced metabolizer). Larger odds ratio for *CYP2B6* and *CYP2A6* in combination relative to either gene alone suggests that variation in these genes independently and additively contribute to lung cancer risk possibly through preferential enzymatic activation of distinct TSNAs—CYP2B6 has a high affinity for NNK, whereas CYP2A6 has a high affinity for NNN [8,9]. Comparable odds ratios for the combination of *CHRNA5-A3-B4* with either *CYP2B6* or *CYP2A6* is also of note and may suggest that concomitant variation in a nicotine/nitrosamine pharmacokinetic and pharmacodynamic gene confers a consistent degree of lung cancer risk.

Combined variation in both *CYP2B6* and *CYP2A6* appeared to confer a greater increase in lung cancer risk (odds ratio of ~3.00) compared to variation in either *CYP2B6* or *CYP2A6* paired with variation in *CHRNA5-A3-B4* (odds ratio of ~2.00). Similarly high odds ratio have been reported for the combined effect of variation in two genes encoding enzymatic activators of polycyclic aromatic hydrocarbons, *MPO* and *CYP1A1* (odds ratio of 2.88) [22]. Genetic variation in multiple pharmacokinetic genes, particularly if activating different procarcinogens, presumably increases the load of activated procarcinogens from cigarette smoke leading to increased DNA adducts and perhaps an increased occurrence of DNA mutations that result in cellular transformation. While there have been numerous studies on the association of gene variants within cigarette smoke pharmacokinetic pathways and lung cancer, less is known regarding variation in pharmacodynamic pathways such as nicotinic receptor signaling, and it remains to be determined whether the combination of gene variants within pharmacokinetic and dynamic pathways will confer a degree of risk similar to that observed in this study.

The combined impact of variation in all three genes is presented in Figure 1C. We separated individuals into four groups based on the number of high-risk genotypes that they possessed: those with none of the high-risk genotypes (0 risk), any one (1 risk), any two (2 risk), or all three high-risk genotypes (3 risk). The 0 risk group served as the reference group. The odds ratios increased in magnitude with each increase in the number of high-risk genotypes. A post-estimation Wald test for homogeneity between the combined genotype groups (*p* = 0.06) also suggests that separating ever-smokers by their combined *CYP2B6/CYP2A6/CHRNA5-A3-B4* genotypes stratified them by their risk for developing lung cancer.

To gain further insight into the mechanisms by which these gene variants could be contributing to lung cancer risk within the context of smoking, we performed subgroups analyses by daily cigarette use and by years of smoking. We previously noted that the impact of *CYP2A6* gene variants was greater on lung cancer risk in the lighter-smoking half of this study population (cigarettes per day ≤20) [1]. We again performed a median split based on cigarette consumption (cigarettes/day ≤20 *vs.* >20) and findings in the lighter-smoking stratum are presented in Figure 1D. Similar to *CYP2A6*, the impact (odds ratio) of *CYP2B6* was larger in the lighter-smoking stratum although it was not statistically significant. When we concurrently evaluated all three genes in a multivariate logistic regression model there was a negligible shift in the odds ratio for each gene (1.54 to 1.56 for *CYP2B6*; 1.65 to 1.64 for *CYP2A6*; 1.58 to 1.60 for *CHRNA5-A3-B4*) again suggesting that variation in each gene is contributing independently to the risk of lung cancer. Combined gene analyses also yielded larger odds ratios in the lighter-smoking stratum (data not shown). Gene effects at lower cigarette exposure have been reported for other metabolic genes such as *CYP1A1*, *GSTM1* and *MPO*[23–25]. At high cigarette (carcinogen) exposure, subtle differences in nitrosamine activation based on genotype may be difficult to detect [26]. Of note, mean daily cigarette consumption within the lighter-smoking stratum was 16.6, which is similar to the reported mean of 15.1 among daily smokers in the US in 2010 [27]. Thus, risk estimates and risk factors among the lighter-smoking stratum in our study population are relevant to present day smoking habits.

We also performed a median split by smoking duration (years of smoking ≤38 *vs.* >38) to gain insight into the temporal relationship between genetic risk factors and carcinogenesis (data not shown). We observed nominally larger odds ratios for *CHRNA5-A3-B4* in the shorter- *vs.* longer-duration stratum, 1.66 *vs*. 1.27, consistent with the association of variation in this chromosomal region with an earlier age of cancer onset [28]. For *CYP2B6* and *CYP2A6*, we observed nominally larger odds ratios in the longer- *vs.* shorter-duration stratum, 2.05 *vs*. 0.75 and 1.66 *vs*. 1.03, respectively. Both enzymes activate nitrosamines into reactive species capable of forming DNA adducts [2,6,8] and perhaps larger odds ratios in the longer-duration stratum reflect increasing cancer risk through an accumulation of carcinogenesis-promoting genotoxic events. The influence of *CYP2B6* genotype group among those smoking for a longer duration appeared to be relatively larger than for *CYP2A6* genotype group. However, it bears noting that this genetic association study was not powered for multiple subgroups analyses.

Based on our results implicating *CYP2B6* and *CYP2A6* gene variants with the risk of developing lung cancer risk among smokers, we hypothesize that altered nitrosamine enzymatic activation contributes to the molecular mechanism of lung carcinogenesis. Evidence corroborating our interpretation include human data demonstrating that CYP2A6 inhibition in cigarette smokers was associated with reduced activation of NNK as indicated by the increased routing of NNK to the metabolite NNAL [29], and by mouse data demonstrating that inhibition of mouse CYP2A enzymes reduced the occurrence of NNK-induced adenomas [30]. Variation in CYP2A13 may also contribute to differences in nitrosamine activation and resulting cancer risk among cigarette smokers—CYP2A13 is predominantly expressed in the respiratory tract and, like CYP2B6, has a high affinity for NNK while playing a negligible role in peripheral nicotine metabolism [31,32]. A number of *CYP2A13* gene variants have been discovered [33], and the CYP2A13*2 allele was associated with lung cancer risk among Chinese [34]. *CYP2A13* was not genotyped within this sample due to the low prevalence of gene variants among European Americans [33].

There are a number of limitations to this study that are inherent to most genetic association studies investigating cancer risk. For example, odds ratio adjustments for cigarette exposure relied on cigarette pack-years, which was derived from self-reported average daily cigarette consumption and years of smoking and may not adequately capture inter-individual differences in carcinogen exposure over time. Statistical power was also a limitation, as anticipated based on results from our previous investigation of *CYP2A6*, which only reached significance in the lighter-smoking stratum [1]. Assuming a comparable effect size for *CYP2B6*, even analyses within the lighter-smoking stratum would be underpowered due to the smaller size of the reference *CYP2B6* genotype group, CYP2B6*6/*6. Thus, reported findings were meant to be mainly investigative (hypothesis generating) in nature but strongly support further investigation of *CYP2B6*, particularly in smoking populations characterized by a higher prevalence of *CYP2B6* gene variants and by lighter-smoking, such as African Americans and Alaska Native people [15,35]. Both African American and Alaska Native smokers experience more difficulty quitting despite lower cigarette consumption [36–39], and it was among the longer-duration and lighter-smokers where we observed larger odds ratio for *CYP2B6* and *CYP2A6*. Among these populations, which have a disproportionately high lung cancer risk [40,41], *CYP2B6* genotype may facilitate the identification of sub-groups of smokers at higher and lower cancer risk.

## 3. Experimental Section

We investigated the association of *CYP2B6* gene variants with lung cancer risk in the case-control population in which we previously investigated *CYP2A6* and *CHRNA5-A3-B4*[1] enabling us to account for the influence of *CYP2A6* and *CHRNA5-A3-B4*, which we already knew to be associated with both cigarette consumption and lung cancer risk. Briefly, all study participants were current or former smokers of European descent. Healthy control subjects were frequency matched to non-small cell lung cancer case subjects by age, gender and smoking variables [1]. This study was approved by Review Boards at the M.D. Anderson Cancer Center (Houston, TX, USA) and the University of Toronto (Toronto, ON, Canada).

For the current analyses, we genotyped participants for the CYP2B6*6 allele, a haplotype defined by the co-occurrence of non-synonymous single nucleotide polymorphisms in exons 4 (G516T) and 5 (A785G) [15,16]. The CYP2B6*6 PCR-based genotyping assay begins with a gene-specific amplification followed by a haplotype-specific amplification in which the co-occurrence of the exon 4 and 5 variants is detected using a forward primer targeted to the exon 4 variant and a reverse primer targeted to the exon 5 variant [16]. The CYP2B6*6 haplotype is prevalent in all world populations ranging in frequency from approximately 14% to 62% [15] and is associated with reduced protein expression and reduced *in vivo* activity for a number of substrates [11,15,42]. We previously genotyped participants for *CYP2A6* alleles with reduced activity that are common in populations of European descent (CYP2A6*2, *4, *9, *12) and for the SNP rs1051730 G>A, which tags the loci associated with both smoking and lung cancer risk within the *CHRNA5-A3-B4* cluster on chromosome 15q25.1 [1,43].

Odds ratios were estimated using logistic regression modeling, which permitted the concurrent evaluation of potential covariates such as cigarette pack-years. Reported odds ratios were adjusted for age, gender and log cigarette pack-years, a common proxy for cigarette exposure. We also adjusted odds ratios for cigarette consumption and years of smoking, separately and concurrently, but observed negligible shifts in the results. In all lung cancer risk analyses, the low-risk genotype group served as the reference group. Statistical analyses were performed with Stata Release 11 (StataCorp LP, College Station, TX, USA).

## 4. Conclusions

*CYP2B6* gene variants are biologically positioned to influence the risk of developing lung cancer among cigarette smokers through altered enzymatic activation of nitrosamines. However, based on reports of genetic linkage and co-regulation with *CYP2A6*, coupled with a potential redundancy of function through substrate overlap, it was unclear whether *CYP2B6* would influence lung cancer risk independently of *CYP2A6*. Furthermore, both enzymes are expressed within the lung and localized nitrosamine activation has been linked to nitrosamine-induced lung tumors [2,4,7].

We found evidence that variation in *CYP2B6* influences lung cancer risk with a similar magnitude to *CYP2A6 and* that variation in these genes influences lung cancer risk independently. Preferential enzymatic activation of distinct TSNAs, NNK by CYP2B6 and NNN by CYP2A6 [8,9], may underlie the observed independent contributions of these genes to lung cancer risk. Combined analyses of concurrent variation in *CYP2B6*, *CYP2A6* and *CHRNA5-A3-B4* appeared to stratify ever-smokers by lung cancer risk supporting an important role for variation in both nicotine/nitrosamine pharmacokinetic and pharmacodynamic pathways to the risk for lung cancer among smokers.

Based on our findings, lung cancer genetic association studies among ethnic populations with a higher percentage of *CYP2B6* gene variants would be beneficial to validate our observations. Furthermore, additional animal studies modulating the expression and/or activity level of nitrosamine metabolizing enzymes, such as CYP2A and CYP2B, would provide valuable insight into the biological mechanism behind our observed differences in lung cancer risk by *CYP2B6* and *CYP2A6* gene variants.

## Supplementary Information



## Figures and Tables

**Table 1:** Characteristics of study subjects and genotyping results.

	Cases, *n* = 398	Controls, *n* = 421	*p* value
Mean Age (sd)	61.9 (10.7)	61.4 (7.4)	0.20
Sex, *n* (%)			
Male	236 (59)	246 (58)	0.80
Female	162 (41)	175 (42)	
Smoking Status, *n* (%)			
Current	206 (52)	196 (47)	0.14
Former	192 (48)	225 (53)	
FTND nicotine dependence score (sd) a	4.81 (2.28)	4.81 (2.56)	0.96
No. cigarettes per day (sd)	27.4 (13.2)	26.8 (14.6)	0.27
Years smoked (sd)	36.9 (12.3)	35.6 (11.8)	0.22

CYP2B6*6 genotyping, *n* (%)			
*1/*1	221 (56)	240 (57)	0.71
*1/*6	157 (39)	156 (37)	
*6/*6	20 (5)	25 (6)	

*CYP2A6* genotype groups b, *n* (%)			
*CYP2A6* NM (normal metabolizer)	326 (82)	327 (78)	0.13
*CYP2A6* RM (reduced metabolizer)	72 (18)	94 (22)	

*CHRNA5-A3-B4* genotype groups c, *n* (%)			
*A5-A3-B4* GG or GA	330 (83)	372 (88)	0.03
*A5-A3-B4* AA	68 (17)	49 (12)	

sd: standard deviation;

a FTND scores missing for 4 cases and 2 controls;

b *CYP2A6* RMs are those individuals with one or more reduced/null activity alleles (CYP2A6*2, *4, *9, *12), while *CYP2A6* NMs have none of the alleles for which we genotyped;

c *CHRNA5-A3-B4* genotype represented by tag SNP rs1051730 G>A with the highest risk genotype for smoking intensity and lung cancer risk kept separate. *p* values were calculated with a chi-square test for categorical variables and a Wilcoxon ranksum test for continuous variables.
