# Supplementary material for: Pilot Study of CYP2B6 Genetic Variation to Explore the Contribution of Nitrosamine Activation to Lung Carcinogenesis
Source: Int J Mol Sci. 2013 Apr 16;14(4):8381–92. doi: 10.3390/ijms14048381 (PMC3645749; doi:10.3390/ijms14048381)
Supplement: Supplementary file 1 [file ijms-14-08381-s001.pdf]

Supplementary Information

Table S1. Lung cancer risk by *CYP2B6*, *CYP2A6* and *CHRNA5-A3-B4* genotype group.

**Genotype Group**	**Cases, *n***	**Controls, *n***	**OR (95% CI)** †	**Adjusted OR (95% CI)** ‡
CYP2B6				
**6/*6*	20	25	1.0 (ref)	1.0 (ref)
**1/*1*, **1/*6*	378	396	1.19 (0.65–2.18)	1.25 (0.68–2.30)
CYP2A6				
reduced metabolizer	72	94	1.0 (ref)	1.0 (ref)
normal metabolizer	326	327	1.30 (0.92–1.83)	1.27 (0.90–1.80)
CHRNA5-A3-B4				
rs1051730 GG/GA	330	372	1.0 (ref)	1.0 (ref)
rs1051730 AA	68	49	1.56 (1.05–2.32)	1.56 (1.05–2.33)

OR: Odds ratio; 95% CI: 95% confidence interval; † Odds ratios estimated by logistic regression modeling; ‡ Adjusted for age, gender, log cigarette pack-years.

Table S2. Lung cancer risk by combined genotype group for each gene pair.

**Genotype Group** [a]	**Cases, *n***	**Controls, *n***	**OR (95% CI)** †	**Adjusted OR (95% CI)** ‡
CYP2B6 & CYP2A6				
0 risk genotypes	2	6	1.0 (ref)	1.0 (ref)
1 risk genotype	88	107	2.47 (0.49–12.5)	2.51 (0.49–12.8)
2 risk genotypes	308	308	3.00 (0.60–15.0)	3.03 (0.60–15.2)
0 or 1 risk, pooled [b]	90	113	1.0 (ref)	1.0 (ref)
2 risk genotypes	308	308	1.26 (0.91–1.73)	1.25 (0.91–1.72)
CYP2B6 & A5-A3-B4				
0 risk genotypes	16	23	1.0 (ref)	1.0 (ref)
1 risk genotype	318	351	1.30 (0.68–2.51)	1.39 (0.72–2.70)
2 risk genotypes	64	47	1.96 (0.93–4.11)	2.07 (0.98–4.37)
CYP2A6 & A5-A3-B4				
0 risk genotypes	64	83	1.0 (ref)	1.0 (ref)
1 risk genotype	274	300	1.18 (0.82–1.71)	1.16 (0.80–1.68)
2 risk genotypes	60	38	2.05 (1.22–3.44)	2.02 (1.19–3.43)

OR: Odds ratio; 95% CI: 95% confidence interval; [a] Risk genotype groups: *CYP2B6 *1/*1*, **1/*6*; *CYP2A6* normal metabolizer; *CHRNA5-A3-B4* AA; [b] Pooled those with 0 or 1 risk genotypes due to the small size of the 0 risk genotype reference group; † Odds ratios estimated by logistic regression modeling; ‡ Adjusted for age, gender, log cigarette pack-years.

Table S3. Combined lung cancer risk by genotype group for all three genes.

Genotype Group [a]	Cases, *n*	Controls, *n*	Adjusted OR (95% CI) †	*p* value ‡
CYP2B6, CYP2A6, A5-A3-B4				
0 risk genotypes	2	5	1.0 (ref)	0.06
1 risk genotype	76	97	2.05 (0.39–10.9)	
2 risk genotypes	264	282	2.43 (0.47–12.7)	
3 risk genotypes	56	37	3.94 (0.72–21.5)	
0 or 1 risk genotype, pooled [b]	78	102	1.0 (ref)	0.03
2 risk genotypes	264	282	1.22 (0.87–1.71)	
3 risk genotypes	56	37	1.97 (1.18–3.30)	

OR: Odds ratio; 95% CI: 95% confidence interval; [a] Risk genotype groups: *CYP2B6 *1/*1*, **1/*6*; *CYP2A6* normal metabolizer; *CHRNA5-A3-B4* AA; [b] Pooled those with 0 or 1 risk genotypes due to the small size of the 0 risk genotype reference group; † Odds ratios estimated by logistic regression modeling and adjusted for age, gender, log cigarette pack-years. ‡ Post-estimation Wald test comparing odds ratios for each risk group.

Table S4. Lung cancer risk within the lighter-smoking stratum (cigarettes per day ≤20).

Genotype Group	Cases, n	Controls, n	OR (95% CI) †	Adjusted OR (95% CI) ‡
CYP2B6				
**6/*6*	7	11	1.0 (ref)	1.0 (ref)
**1/*1*, **1/*6*	192	210	1.44 (0.55–3.78)	1.54 (0.58–4.08)
CYP2A6				
reduced metabolizer	40	65	1.0 (ref)	1.0 (ref)
normal metabolizer	159	156	1.66 (1.05–2.60)	1.65 (1.05–2.60)
CHRNA5-A3-B4				
rs1051730 GG/GA	172	201	1.0 (ref)	1.0 (ref)
rs1051730 AA	27	20	1.58 (0.85–2.91)	1.58 (0.85–2.94)

OR: Odds ratio; 95% CI: 95% confidence interval; † Odds ratios estimated by logistic regression modeling. ‡ Adjusted for age, gender, log cigarette pack-years.

© 2013 by the authors; licensee MDPI, Basel, Switzerland. This article is an open access article distributed under the terms and conditions of the Creative Commons Attribution license (http://creativecommons.org/licenses/by/3.0/).
